# Optimal method for metabolic tumour volume assessment of cervical cancers with inter-observer agreement on [18F]-fluoro-deoxy-glucose positron emission tomography with computed tomography

**DOI:** 10.1007/s00259-020-05136-8

**Published:** 2020-12-11

**Authors:** Mubarik A. Arshad, Samuel Gitau, Henry Tam, Won-Ho E. Park, Neva H. Patel, Andrea Rockall, Eric O. Aboagye, Nishat Bharwani, Tara D. Barwick

**Affiliations:** 1 Departments of Radiology and Nuclear Medicine, Hammersmith Hospital, Imperial College Healthcare NHS Trust, Du Cane Road, London, W12 0HS UK; 2 Department of Clinical Oncology, Hammersmith Hospital, Imperial College Healthcare NHS Trust, Du Cane Road, London, W12 0HS UK; 3 Department of Surgery & Cancer, Hammersmith Hospital, Imperial College London Cancer Imaging Centre, Du Cane Road, London, W12 0NN UK

**Keywords:** Cervix, FDG, PET/CT, MTV, Tumour segmentation

## Abstract

**Purpose:**

Cervical cancer metabolic tumour volume (MTV) derived from [18F]-FDG PET/CT has a role in prognostication and therapy planning. There is no standard method of outlining MTV on [18F]-FDG PET/CT. The aim of this study was to assess the optimal method to outline primary cervical tumours on [18F]-FDG PET/CT using MRI-derived tumour volumes as the reference standard.

**Methods:**

81 consecutive cervical cancer patients with pre-treatment staging MRI and [18F]-FDG PET/CT imaging were included. MRI volumes were compared with different PET segmentation methods. Method 1 measured MTVs at different SUV_max_ thresholds ranging from 20 to 60% (MTV_20_-MTV_60_) with bladder masking and manual adjustment when required. Method 2 created an isocontour around the tumour prior to different SUV_max_ thresholds being applied. Method 3 used an automated gradient method. Inter-observer agreement of MTV, following manual adjustment when required, was recorded.

**Results:**

For method 1, the MTV_25_ and MTV_30_ were closest to the MRI volumes for both readers (mean percentage change from MRI volume of 2.9% and 13.4% for MTV_25_ and − 13.1% and − 2.0% for MTV_30_ for readers 1 and 2). 70% of lesions required manual adjustment at MTV_25_ compared with 45% at MTV_30_. There was excellent inter-observer agreement between MTV_30_ to MTV_60_ (ICC ranged from 0.898–0.976 with narrow 95% confidence intervals (CIs)) and moderate agreement at lower thresholds (ICC estimates of 0.534 and 0.617, respectively for the MTV_20_ and MTV_25_ with wide 95% CIs). Bladder masking was performed in 86% of cases overall. For method 2, excellent correlation was demonstrated at MTV_25_ and MTV_30_ (mean % change from MRI volume of −3.9% and − 8.6% for MTV_25_ and − 16.9% and 19% for MTV_30_ for readers 1 and 2, respectively). This method also demonstrated excellent ICC across all thresholds with no manual adjustment. Method 3 demonstrated excellent ICC of 0.96 (95% CI 0.94–0.97) but had a mean percentage difference from the MRI volume of − 19.1 and − 18.2% for readers 1 and 2, respectively. 21% required manual adjustment for both readers.

**Conclusion:**

MTV_30_ provides the optimal correlation with MRI volume taking into consideration the excellent inter-reader agreement and less requirement for manual adjustment.

**Supplementary Information:**

The online version contains supplementary material available at 10.1007/s00259-020-05136-8.

## Introduction

Cervical cancer, the fourth most common gynaecological malignancy worldwide, is a major cause of mortality in women [1, 2]. Primary tumour volume–derived parameters from ^18^F-fluoro-deoxy-glucose positron emission tomography with computed tomography ([18F]-FDG PET/CT) such as metabolic tumour volume (MTV) and total glycolytic volume (TGV) have been reported to be prognostic in cervical cancer patients [3–5]. Combined nomograms of pre-treatment MTV, cervical tumour maximum standard uptake value (SUV_max_) and lymph node status on [18F]-FDG PET/CT have been suggested to predict overall survival in locally advanced cervical cancer patients undergoing chemo-radiation therapy [6].

Even though morphological MRI has assumed prominence as the imaging modality of defining the gross tumour volume (GTV) in cervical cancer adaptive brachytherapy, as enshrined in Gynaecological European Group of Curietherapie-European Society for Therapeutic Radiology and Oncology (GYN GEC-ESTRO) Working Group recommendations, PET retains a role in staging (N, M) and prognosis and is integrated into the radiotherapy workflow [7–9].

However, the optimal method of outlining tumour volume on [18F]-FDG PET/CT in cervical cancer has not been established, and this is required in order to standardise its use for establishing prognosis using volumetric based parameters (Table 1).

**Table 1:** Select cervical cancer studies with FDG PET and the threshold chosen

PET cervix volumes, Authors/Paper	Year	Number of patients	Comparison with/gold standard	Findings	Software used for PET outline	Threshold used/recommended
Miller, Grigsby. *Journal of Rad Onc*. [10]	2002	13	PET only scanner compared with CT scan (unenhanced)	Manual adjustment in ¼ of cases	Not stated	40% threshold
Ho et al., *EJNMMI* [11]	2009	33	T2 and ADC at 3-T manual ROI on axial slices by visual inspection of T2 and ADC map. Tumour volume calculated as the sum of all ROI areas on transaxial multiplied by 5 mm	2 methods: a fixed threshold of 40% the SUV_max_ to approximate cervical tumour volume, the other using best volumetric match or optimal threshold	GE workstation	Optimal thresholds 42.5% ± 8.0% if tumour > 5 cm (*n* = 10), 45.2% ± 6.5% tumour 3–5 cm (*n* = 14), and 51.5% ± 7.7% if tumour < 3 cm (*n* = 9). Difference between 40% threshold and MR tumour volume marginally significant (*p* = 0.0792). Since single 40% threshold could not provide accurate volume, the optimal threshold for each tumour SUV_mean_ measurements were used in subsequent analyses.
Ma, Grigsby, *Radiotherapy and Oncology* [12]	2011	47	1.5-T MRI: sagittal and axial T2W. Manual outline. Exact method not fully described	MRI better visualises larger tumours in reference to FDG PET/CT. FDG PET/CT visualised tumour volumes different from T2-weighted MRI, especially in tumours < 14 cc with regard to location.	Treatment planning workstation (Varian Eclipse Treatment Planning System V6.5)	40% threshold only assessed and underestimated large tumour on MRI
Upasani et al., *International Journal of Gynaecological Cancer* [13]	2012	74 stage IIb and IIIB squamous cancer	Tri-diameter ellipsoid (*V* = *d*1*d*2*d*3*π*/6) MRI T2	Primary tumour volume estimation at 30 to 35% of SUV_max_ values correlated significantly with the criterion standard MR volumes for primary cervical tumour with squamous histology.	GE software	30% or 35% threshold
Sun et al., *EJNMMI* [14]	2014	35	PET/MRI scanner 3-T MRI (T2W and DWI). Both manual outline axial slice × slice profile (5-mm slice thickness plus 1.0-mm intersection gap)	Compared with 20% to 60% SUV_max_ (5% increments)	Phillips Brilliance Workspace	Strong correlation between FDG PET, T2W and DWI-cut off 35% or 40%
Zhang et al., *Nucl Med Comm* [15]	2014	27	Axial T2W manual outline. Exact method not fully described	Volume discrepancies between MR and PET volumes with smaller volumes	Phillips Extended Brilliance Workstation	40%
Lai et al., *BMC Cancer* [16]	2017	29	T2W manual outlines in sagittal and axial oblique multiplied by the slice thickness	MTV_30_ correlated best with the anatomical volume–derived from T2W MR	Advanced volume share on ADW 4.7 workstation (GE Healthcare)	30% threshold
Cegła et al., *Contemp Oncol* [17]	2019	30	MRI-unclear which sequences or method	MTV_35_ most closely matched the MRI volume	Not disclosed	35% threshold

The EANM Guidelines for Tumour Imaging suggest a 41% of the SUV_max_ tumour VOI corresponds best with the tumour dimensions provided the tumour has high metabolic activity to background ratios and homogenous uptake [18]. Otherwise, a VOI of 50% of the SUV_max_ was recommended. These guidelines were based on three papers, a phantom thorax study [19], a test re-test study in 11 lung cancer patients and in the follow-up of 16 breast cancer patients [20], and repeatability measurements in 11 lung cancer patients scanned 7 days apart [21]. All of the studies cited by the guidelines were performed by the same single institution, and those that were performed on humans utilised tumours with high tumour to background ratios (lung and breast). It is unclear, but unlikely, that this automatically extends to other tumour types particularly tumours close to organs with high physiologic activity such as cervical tumours adjacent to the bladder.

An early study by Miller and Grigsby involving 13 patients with cervical cancer who had [18F]-FDG PET within 2 weeks of separately acquired CT established a threshold of 40% SUV_max_ (MTV_40_) based on the visual inspection of tumour volume on CT scans [10].

Most subsequent studies (Table 1) on cervical tumours used this MTV_40_ threshold [6, 11, 15, 22–24], although a few studies have used a fixed threshold of SUV > 2.5 [3, 5, 25]. Recent studies have suggested 30% SUV_max_ (MTV_30_) or 35% SUV_max_ (MTV_35_) threshold correlate best with tumour volume on MRI [13, 16]. In tumour volume assessment of other malignancies, a fixed SUV_max_ threshold has demonstrated significant limitations, including underestimating MTV in lesions with high SUV_max_ and overestimating in lesions close to regions with high background activity [26]. In addition, partial volume averaging affects small tumours. Recently, automated gradient methods have been proposed but to date, they have not been assessed in cervical tumours [27].

Ideally, the tumour segmentation technique should be fully automated. However, in practice, intense bladder/ureteric and bowel activity adjacent to the cervical tumour can interfere with accurate tumour outlining. Therefore, manual adjustment of automated volumes, to exclude activity in adjacent physiologic structures, may be required. This has been mentioned but not accurately documented in previous studies [16]. In addition, it is not clear which software approach to exclude bladder is best and options include bladder masking or applying a constraining volume around the tumour prior to applying thresholds.

With the increasing trend towards volumetric assessment of PET data with radiomics feature analysis, it is important that the methods are standardised using a technique that is both accurate and reproducible. The reproducibility of cervical tumour volume assessment on [18F]-FDG PET/CT at various thresholds has never previously been documented.

The aims of this study are as follows:

1. To evaluate the optimal metabolic tumour volume (MTV) at different percentage rates of SUV_max_ thresholds (method 1 using bladder masking when required; method 2 using an ellipsoid isocontour around the tumour before applying thresholds) and an automated gradient method (method 3) to outline primary cervical tumours using MRI-derived tumour volumes as to the reference standard.
2. To document any requirement for manual adjustment.
3. To assess inter-observer agreement in MTV measurement.

## Method

### Study design

Institutional ethical approval for retrospective analysis was obtained, and informed consent was waived. Consecutive patients between January 2009 and December 2016 who had staging [18F]-FDG PET/CT imaging for biopsy-proven cervical cancer at our tertiary referral specialist gynaecological oncology centre and MRI pelvis were included in the study. Inclusion criteria were (i) histologically confirmed cervix cancer, (ii) absence of previous treatment for cervical cancer (including previously excised by cone biopsy), (iii) availability of a recent comparative MRI pelvis within 10 weeks and (iv) presence of a measurable cervical tumour on both MRI and PET/CT. Exclusion criteria were as follows: patients in whom the cervical tumour was not measurable (less than 5 ml in volume [28, 29]).

### PET/CT protocol

The PET centre is NCRI (National Cancer Research Institute) accredited by the UK PET Core Lab and all scans were performed on the same scanner (Siemens Biograph 64). Following a 4–6-h fast with acceptable glucose level (< 11 mmol/l) patients were administered ^18^F-FDG (370–410 MBq) intravenously. Post 60-min uptake period, a low-dose CT (5-mm thickness with 3-mm spacing, 120 kVp, 50 mAs, 0.8 spiral pitch) was performed followed by an emission study (mid-thighs to skull base, 5–6 overlapping bed positions 3–4 min/bed position). Images were reconstructed using ordered subset expectation maximisation (4 iterations, 8 subsets, Gaussian filter of 5 mm FWHM). The PET images were attenuation-corrected using the CT data.

### Segmentation

#### Method 1: Semi-automated adaptive threshold contour generation ± bladder masking

##### Bladder masking

For each MTV threshold, an initial assessment was made whether bladder masking was required. The criterion for bladder masking was as follows: if the bladder was outlined instead of tumour or if part of the bladder was included in the MTV on > 5 slices. When required a single experienced observer (SG) performed bladder masking using an automated technique (Hermes Medical Solutions, Sweden).

##### Image analysis

Images were analysed independently by two experienced observers (SG and TB, 3- and 15-years’ PET/CT experience respectively). MTV was auto-contoured at percentage SUV_max_ thresholds of 20%, 25%, 30%, 35%, 40%, 50% and 60% (MTV_20, 25, 30, 35, 40, 50_ and _60_) (Hermes Medical Solutions, Sweden). Percentage SUV_max_ thresholds were performed at intervals of 5% from 20 to 40% based on the findings of the study by Upasani et al. [13], which suggested that in their cohort the ideal threshold was between MTV_30_ and MTV_35_ and by earlier research which suggested a MTV_40_ threshold [10]. In addition, an absolute threshold of SUV > 2.5 (SUV_2.5_), as used by other studies, was assessed [3, 25]. If adjacent physiological structures, such as bladder, ureters and bowel, were included in the automated volume, manual adjustments were made. The degree of manual adjustment was documented as either no manual adjustment, minor: ≤ five slices, major: six slices up to twenty, too difficult: 21 slices or more to adjust. In the few cases deemed ‘too difficult to employ manual adjustment’, the MTV was not measured and this was noted.

The MTV for the different thresholds was recorded for each observer. The inter-observer agreement for MTV was assessed from the final volume for each observer, i.e. included completely automated cases and those with manual adjustment if required.

#### Method 2: Semi-automated adaptive threshold contour generation with isocontour method

Percentage SUV_max_ outlining was also performed using an ellipsoid isocontour method (Siemens Syngo.Via, Siemens Healthineers AG, Germany) independently by two experienced observers (MA and TB, 5- and 15-years’ PET/CT experience respectively). An ellipsoid contour was drawn around the tumour avoiding adjacent physiologic structures and negating the need for bladder masking. This then constrained the volume from which the different thresholds were obtained. The isocontours were drawn separately by the two observers. No manual adjustment of the final tumour thresholds was possible with this software.

#### Method 3: Automated gradient method

Using the Automated Gradient–based method (MIM Software Inc., Cleveland OH, USA), which places the contour boundary at the location where the signal gradient is the greatest [30], two observers (TB and MA) produced segmentations. The technique required the observer to select the tumour with two perpendicular cross hairs which then generated the segmentation. Any adjacent structures which were outlined but were not related to the tumour were manually removed. The same manual adjustment scoring system used for method 1 was again utilised. Bladder masking was also utilised if required.

For each method, the observers received training in ten random cases with the application specialist.

##### MRI pelvis protocol and image analysis

Staging MRI pelvis was performed at the local network hospital or the tertiary centre. As such, there was variation in MRI scanner and exact protocol. However, all scans were performed at 1.5 T and as a minimum included 2D small field of view T2-weighted (T2W) sequences in sagittal and axial oblique (perpendicular to the long axis of the cervix) planes. The maximum slice thickness was 5 mm across the network as per GYN GEC-ESTRO (at the tertiary centre sagittal T2 MRI parameters: turbo-spin echo, TR 2275 ms, TE 90 ms, voxel size 0.57/0.57/5.0 mm, thickness/interval 5 mm/0 mm). Additional sequences included T1-weighted and diffusion-weighted images.

A single radiologist (NB, 12 years’ experience pelvic MR imaging) outlined the cervical tumours on MRI on the sagittal T2W sequence [31]. The reader manually contoured around the outer edge of the cervical tumour on each image correlating with other sequences as necessary. The total tumour volume was automatically calculated as the sum of each of the cross-sectional volume measurements (cross-sectional area multiplied by section thickness) [16, 32]. Where the primary tumour contained regions of necrosis centrally, these were included in the volume measurement. Care was taken to avoid the inclusion of adjacent normal tissue in the region of interest (ROI).

### Statistical analysis

Statistical analysis was performed using SPSS (SPPS v22, IBM, New York, US). The MRI and PET/CT volumes at different thresholds were correlated using scatterplots and Pearson correlation test. Correlation is the extent to which 2 or more variables are associated with each other and the strength of the relationship is assigned an *r* value. Correlation and percentage difference of each MTV_*x*%_ on PET/CT with the MRI volume was made. Correlation *r* values were scored as follows: small 0.1 to 0.29, medium 0.3 to 0.49 and large 0.5 to 1.0 [33]. A *p* value < 0.05 was considered to indicate statistical significance.

Inter-observer variability in the volume measurements at each threshold, following manual correction if required, was assessed using the intraclass correlation coefficient (ICC) estimates along with their 95% confidence intervals using a two-way random absolute single measures model. Values less than 0.5, between 0.5 and 0.75, between 0.75 and 0.9, and greater than 0.90 are indicative of poor, moderate, good and excellent reliability, respectively [34].

The paired *t* test statistical technique was used to compare the independent readings between reader 1 and reader 2. To compare each reader to the MRI volumes, an independent *t* test was used. A 2-tailed paired *t* test with 95% CI was also used to compare each MTV threshold for the separate readers with the MRI volume. Bland-Altman plots were used to visually assess the distribution of differences (spread of points along *y*-axis) and to compare the distribution of estimates obtained for segmentations (spread and separation of points along *x*-axis). In addition, correlation between low, intermediate and high SUV_max_ for select MTV thresholds and the presence or absence of necrosis was made with the MRI volume.

## Results

### Study population

Between January 2009 and December 2016, 118 patients with cervical cancer underwent staging PET/CT imaging at our institution. Eleven patients were excluded as they had been treated previously, 14 for lack of corresponding staging MRI pelvis and 12 for inability to perform volume measurements at the different MTV thresholds due to their very small volume (less than 5 ml [28, 29]). 81 patients were therefore included in this study. The time interval between the staging MR and PET/CT was a mean of 16.2 days (range 0–64 days).

38% of patients had FIGO [35] stage IIB disease (Table 2). The mean primary tumour volume was 85.4 cm^3^ on MRI (range 6.7–413). The mean SUV_max_ of the primary tumour was 15.1 (± 6.9 SD). The mean and range of SUV_max_ according to histological subtype is presented in Table 2.

**Table 2:** Baseline characteristics

**Age (years)**	**Mean**	**Range**		
	48.8	24.9–89.7		
**FIGO stage**	**N**	**%**		
IB	13	16.1		
IIA	9	11.1		
IIB	31	38.3		
IIIA	5	6.2		
IIIB	10	12.3		
IVA	7	8.6		
IVB	6	7.4		
**Primary tumour MRI volume and SUV**_**max**_	**Mean**	**SD**	**Median**	
Primary tumour MRI volume (ml)	85.4	69.8	74.4	
Primary tumour SUV_max_	15.1	6.9	14.3	
Histology *	**N**	**%**	**SUV**_**max**_ **mean**	**Range**
Adenocarcinoma	10	12.3	15.1	6.17–26.1
Squamous cell	65	80.2	15.1	6.0–50.3
Adenosquamous	4	4.9	16.5	8.28–22.97
Neuroendocrine **	2	2.5	12.1	11.3–12.9

FIGO staging systems are determined by the International Federation of Gynaecology and Obstetrics (Fédération Internationale de Gynécologie et d’Obstétrique)

*There was no significant difference between histological subtype and mean SUV_max_

**Both were poorly differentiated

### Bladder masking

**Fig. 1:**
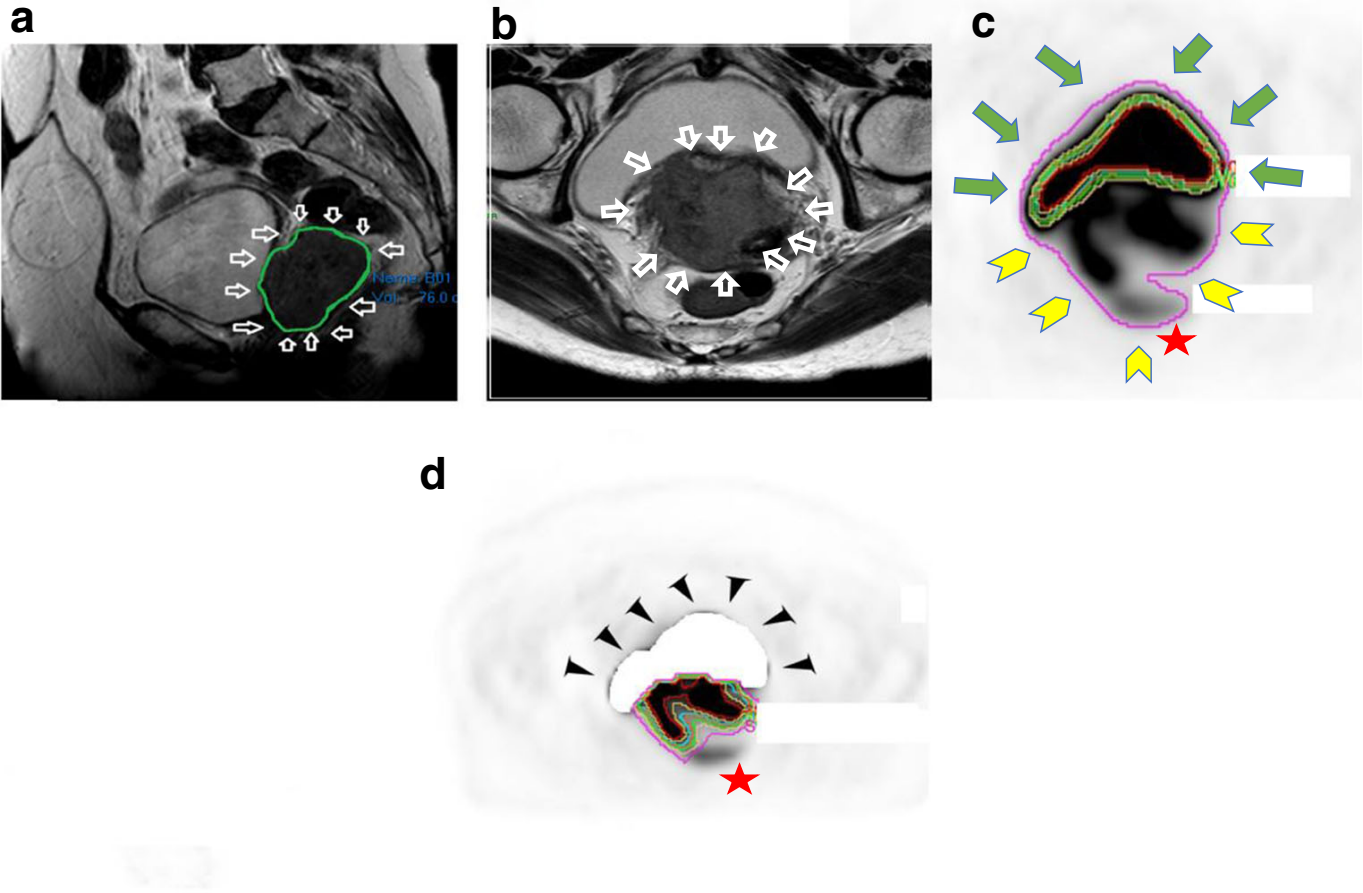
Tumour segmentations on MRI and PET using method 1. **a** Sagittal (green outlining segmentation) and arrow. **b** Axial (white arrows) T2W. **c** Axial [18F]-FDG PET without bladder masking and automatic thresholding at fixed SUV thresholds demonstrating the bladder (bladder-anterior green arrows; tumour posterior—yellow chevrons being selected over the tumour at most of the MTV thresholds. Star denotes that at some thresholds the bowel was inadvertently outlined). **d** [18F]-FDG PET axial with bladder masking demonstrated tumour segmentations at various thresholds. Star denotes no bowel segmentation. For this patient: MRI volume 76 ml, SUV_2.5_ (pink) 109.2 ml, MTV_25_ (beige) 85.9 ml, MTV_30_ (green) 75.4 ml, MTV_35_ (orange) 68.7 ml, MTV_40_ (turquoise) 61.8 ml, MTV_50_ (yellow) 45.6 ml, MTV_60_ (red) 29.2. In this example, the MTV_30_ threshold was the closest to the MRI volume

For method 1, bladder masking prior to auto-contouring was performed on 86% of patients (Fig. 1 and Table 3). This was dependent on the MTV threshold used with the greater requirement at lower thresholds. At MTV_60_, 61 (75.3%) of PET scans required masking, which increased to 77 (95.1%) at MTV_20_. For method 2, no bladder masking was performed as this method constrains an elliptical volume avoiding bladder (where possible). For method 3, bladder masking was performed in 4% (Table 3).

**Table 3:** The number requiring bladder masking at different PET thresholds for methods 1 and 3. For method 1, overall 86% required masking and 14% did not require bladder masking. For method 3, 96% did not require bladder masking

	Method 1							Method 3
Threshold	MTV_60_	MTV_50_	MTV_40_	MTV_35_	MTV_30_	MTV_25_	MTV_20_	
*N*	81	81	81	81	81	81	81	76
Required bladder masking *n*, (%)	61 (75.3)	63 (77.8)	68 (84)	71 (87.7)	72 (88.9)	75 (92.6)	77 (95.1)	3 (4)
Did not require bladder masking *n*, (%)	20 (24.7)	18 (22.2)	13 (16)	10 (12.3)	9 (11.1)	6 (7.4)	4 (4.9)	73 (96)

### Manual adjustment at different thresholds

The requirement for manual adjustment of the auto-contoured volumes at each SUV_max_ threshold for method 1 is documented in Table 4. For example, the MTV_25_ required 69% adjustment (of which minor 26%, major 35%, too difficult 7.5%) and MTV_30_ thresholds needed 44% adjustments (minor 17%, major 25%, too difficult 2.5%). At MTV_40_ threshold, there was only 22% adjustment (minor 11%, major 10%, too difficult 1.2%), whilst at MTV_60_, only 4% required adjustment (minor 2.5%, major 1.25%). For method 2, no manual adjustment was possible once the isocontour was selected. For method 3, overall 23.7% required adjustment (minor 2.6%, major 19.7%, too difficult 1.3%) (Table 4).

**Table 4:** Adjustments for methods 1 and 3. None: No manual adjustment, minor: ≤ five slices, major: 6–20, too difficult > 21 slices

Threshold	None	Minor	Major	Too difficult	Total	Threshold
Method 1						
SUV_2.5_	4	2	72	3	81	SUV_2.5_
MTV_20_	18	24	32	7	81	MTV_20_
MTV_25_	25	21	29	6	81	MTV_25_
MTV_30_	45	14	20	2	81	MTV_30_
MTV_35_	53	13	14	1	81	MTV_35_
MTV_40_	63	9	8	1	81	MTV_40_
MTV_50_	70	9	2	0	81	MTV_50_
MTV_60_	78	2	1	0	81	MTV_60_
Method 3						
Reader 1	59	1	14	1	76	Reader 1
Reader 2	58	2	14	1	76	Reader 2

The previous submitted table had some missing data for the manual data which we have retrieved so that there is no missing data

### Correlation between tumour volumes on MRI and PET

Tumour volumes as measured on MRI and at different MTV thresholds are summarised in Table 5. There was a large positive correlation between MRI volume and MTV_20–60_ and no correlation with MRI volume and SUV_2.5_.

**Table 5:** Mean tumour volume measurements on MRI and [18F]-FDG PET/CT using the 3 methods. Correlation between MRI volume and MTV at each threshold. Difference between mean MRI volumes and MTV. The mean percentage change from the MRI volume is also given

Threshold	Reader	Mean (ml)	Standard deviation	Median (ml)	N	Pearson correlation, *r*	Mean MRI volume and MTV difference (paired *t* test with 95% CI)	Mean percentage difference from MRI
MRI		85.4	69.8	74.4	81			
Method 1								
MTV_2.5_	Reader 2	267.6	1099	125.3	80	− 0.057		69.7
MTV_20_	Reader 1	97.2	64.3	89.8	78	0.645	0.075 (− 1.12 to 22.8)	13.82
	Reader 2	66.7	69.3	51.7	81	0.508	0.017 (− 33.9 to − 3.4)	32.8
MTV_25_	Reader 1	87.9	66.9	79.4	80	0.801	*0.599* (− 7.0 to 12.1)	2.93
	Reader 2	80.0	56.6	77.7	81	0.653	*0.374* (− 17.3 to 6.6)	13.4
MTV_30_	Reader 1	74.3	57.5	68.6	80	0.784	0.023 (− 20.8 to − 1.58)	− 13.11
	Reader 2	73.3	54.9	66.0	81	0.789	0.013 (− 21.6 to − 2.6)	− 2.0
MTV_35_	Reader 1	62	44.9	54.8	81	0.764	0.005 (− 33.5 to − 13.2)	− 27.40
	Reader 2	63.7	46.2	56.0	81	0.770	0.005 (− 31.7 to-11.7)	− 17.2
MTV_40_	Reader 1	52	38.5	45	80	0.712	0.005 (− 44.4 to − 22.2)	− 39.11
	Reader 2	53.1	40.6	46.2	81	0.726	0.005 (− 43.1 to − 21.4)	− 31.1
MTV_50_	Reader 1	36.4	28.4	31	79	0.609	0.005 (− 61.6 to − 36.3)	− 57.38
	Reader 2	37.8	28.8	33.2	81	0.655	0.005 (− 59.8 to − 35.3)	− 50.6
MTV_60_	Reader 1	23.2	18.9	18.9	78	0.387	0.005 (− 76.5 to − 47.3)	− 72.83
	Reader 2	24.4	18.7	21.9	81	0.529	0.005 (− 74.7 to − 47.3)	− 67.7
Method 2								
SUV_2.5_	Reader 1	109.0	82.9	93.4	81	0.849	0.005 (− 33.3 to − 14.0)	27.6
	Reader 2	104.2	70.8	87.0	81	0.82	0.005 (− 28.3 to − 9.64)	22
MTV_20_	Reader 1	95.2	68.1	84.3	81	0.829	0.032 (− 18.7 to − 0.89)	11.5
	Reader 2	88.6	58.9	78.2	81	0.810	*0.473* (− 12.4 to 5.79)	3.75
MTV_25_	Reader 1	82.1	57.3	74.9	81	0.848	*0.434* (− 4.96 to 11.4)	− 3.9
	Reader 2	78.1	51.0	69.1	81	0.836	*0.097* (− 1.36 to 15.9)	− 8.6
MTV_30_	Reader 1	71.0	48.6	67.4	81	0.844	0.005 (5.81 to 23.0)	− 16.9
	Reader 2	69.2	45.3	63.9	81	0.842	0.005 (7.33 to 25.0)	− 19
MTV_35_	Reader 1	61.3	40.9	55.9	81	0.810	0.005 (14.3 to 33.7)	− 28.2
	Reader 2	59.4	40.4	53.4	81	0.822	0.005 (16.4 to 35.6)	− 30.4
MTV_40_	Reader 1	52.8	35.1	46.9	81	0.762	0.005 (21.8 to 43.3)	− 38.2
	Reader 2	52.5	35.7	46.8	81	0.793	0.005 (22.5 to 43.2)	− 38.5
MTV_50_	Reader 1	38.0	25.1	33.2	81	0.668	0.005 (34.9 to 57.8)	− 55.5
	Reader 2	38.4	26.3	34.1	81	0.745	0.005 (35.2 to 58.7)	− 55
MTV_60_	Reader 1	25.1	17.4	21.3	81	0.553	0.005 (46.6 to 74.0)	− 70.6
	Reader 2	25.7	18.0	22.4	81	0.642	0.005 (46.5 to 72.9)	− 69.9
Method 3								
Gradient	Reader 1	66.6	48.5	58.2	77	0.814	0.005 (9.72 to 29.0)	− 19.14
	Reader 2	67.4	50.1	58.6	77	0.785	0.005 (8.86 to 29.0)	− 18.24

Italics depict no significant difference between the MTV threshold and the MRI volumes

*N* sample size, *r* Pearson’s correlation coefficient

There was no significant difference between the MRI volume and MTV_25_ for both readers. There was a significant difference between MRI volume and the other MTV values (Table 5).

**Fig. 2:**
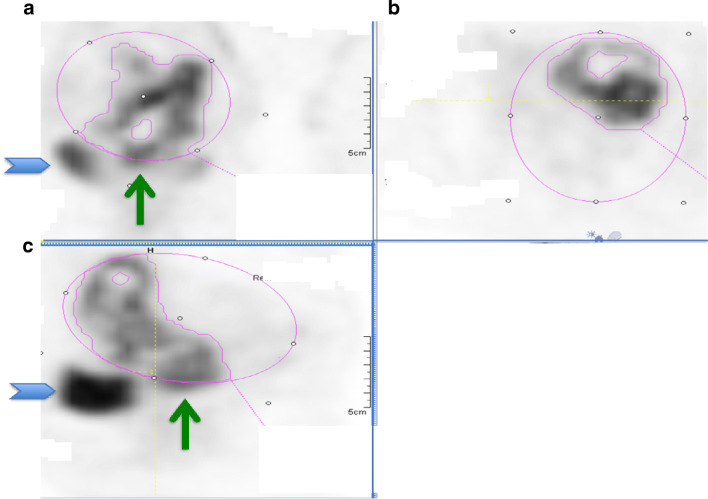
Method 2 ellipsoid isocontour method: FDG PET, **a** coronal view, **b** axial, **c** sagittal. The user encircles the ellipsoid isocontour around the tumour (in pink), and the software segments the tumour within. Different MTV thresholds can be selected. The blue chevron indicates the bladder. The thin green arrow indicates the tumour outside the ellipsoid isocontour, which despite adjustments cannot include the tumour in its entirety and exclude the bladder. This lesion SUV_max_ 15.1, MTV 26.5 ml

For method 1, MTV_25_ and MTV_30_ were closest to the MRI volumes for both readers (mean percentage change from MRI volume of 2.9% and 13.4% for MTV_25_ and − 13.1% and − 2.0% for MTV_30_ for readers 1 and 2 respectively) (Table 5, Fig. 1). For method 2 (ellipse isocontour method), MTV_25_ and MTV_30_ were also closest to the MRI volumes for both readers (mean percentage change from MRI volume of − 3.9% and − 8.6% for MTV_25_ and − 16.9% and − 19% for MTV_30_ for readers 1 and 2 respectively) (Table 5 and Fig. 2). In addition, the best correlation was at the MTV_25_ and MTV_30_ for both readers although there was also excellent correlation with an *r* > 0.75 between MTV_20_ to MTV_40_ and the absolute SUV_2.5_.

The higher thresholds MTV_40_ to MTV_60_ significantly underestimated tumour volumes for both percentage threshold methods. For example, the MTV_40_ had a good positive correlation with the MRI volume, but the volumes were underestimated by a mean of 39.1% for reader 1 and 31.1% for reader 2 for method 1 and 38.2% and 38.5% respectively for method 2.

Using a fixed SUV_2.5_ led to a marked overestimation of tumour size (mean 69.7% overestimation), as adjacent background structures were included in the volume using method 1, whilst method 2 also showed a significant overestimation but less marked as the elliptic isocontour method permits a restraining volume.

For method 3, automated gradient method, there was a good correlation between the PET segmentation and the MRI volume (reader 1: 0.81 and reader 2: 0.79). This method, however, significantly underestimated the tumour volume compared with the MRI (− 19.1 and − 18.2% for readers 1 and 2, respectively).

The impact of tumour SUV_max_ and presence or absence of necrosis on over- or underestimation of MRI volume is presented in Supplementary Figs. 1 and 2.

### Inter-observer agreement

The inter-observer agreement (Table 6) was good to excellent for method 1 for thresholds MTV_30_ to MTV_60_ (ICC estimates ranging from 0.898 to 0.976 with narrow 95% confidence intervals). Inter-observer agreement was moderate at the lower thresholds (ICC estimates of 0.534 and 0.617 respectively for the MTV_20_ and MTV_25_ with wide 95% confidence intervals).

**Table 6:** Inter-observer agreement between reader 1 and reader 2 using the intraobserver class correlation for the three methods

Threshold	Single measure intraclass correlation	95% CI
Method 1		
MTV_20_	0.534	0.30–0.70
MTV_25_	0.617	0.46–0.74
MTV_30_	0.955	0.93–0.97
MTV_35_	0.976	0.96–0.99
MTV_40_	0.947	0.92–0.97
MTV_50_	0.911	0.87–0.94
MTV_60_	0.898	0.85–0.93
Method 2		
SUV_2.5_	0.935	0.901–0.958
MTV_20_	0.898	0.845–0.934
MTV_25_	0.947	0.918–0.966
MTV_30_	0.977	0.965–0.985
MTV_35_	0.960	0.938–0.974
MTV_40_	0.982	0.971–0.988
MTV_50_	0.969	0.953–0.980
MTV_60_	0.973	0.958–0.982
Method 3		
Gradient	0.962	0.942–0.975

For method 2, there was excellent inter-observer agreement across all thresholds from MTV_25_ to MTV_60_ (ICC estimates ranging from 0.935–0.973 with narrow 95% confidence intervals). Although readers could vary in the selection of the isocontour boundaries, no manual adjustments were possible with this method.

For the automated gradient method agreement was excellent (ICC estimate 0.96 (0.942–0.975, 95% CI)).

When Bland-Altman plots were performed (Supplementary Fig. 3), only the MTV_25_ for reader 1 on method 1 demonstrated no proportional bias between the MRI and PET segmentations.

### Optimal threshold

In summary, there was no significant difference between the mean MRI volume and MTV_25_ for both methods 1 and 2. Both MTV_25_ and MTV_30_ were closest to the MRI volume for both readers. The MTV_30_ had excellent inter-observer agreement (ICC between the two readers, *r* = 0.955 (95% CI 0.93–0.97)), compared to *r* = 0.62 (95% CI 0.46–0.74) for MTV_25_, (Table 6). There were fewer requirements for manual adjustment at MTV_30_ compared to MTV_25_ (44% and 70% manual adjustment respectively) using method 1. Using method 2, there was higher correlation between the thresholds of MTV_20_ to MTV_35_, excellent ICC between readers at all thresholds and with no manual adjustment requirement of the tumour VOI. Method 3, the automated gradient method, had excellent observer agreement but significantly underestimated the volume compared to MRI.

However, the presence of necrosis and extremes of SUV_max_ could impact the lower threshold MTVs (Supplementary Figs. 1 and 2), limiting the usage of MTV_25_.

## Discussion

Variations in FDG uptake for different histological subtypes have been previously reported with squamous cell carcinoma (SCC) being the histological type with the highest metabolic intensity and neuroendocrine tumours often presenting a heterogeneous uptake including a well-differentiated neuroendocrine part with no/low uptake [36, 37]. Whilst SCC showed the highest uptake, overall, we did not find a difference between SCC, adenocarcinoma, adenosquamous carcinoma and neuroendocrine histological subtypes (Table 2). It is possible that this resulted from the vast majority (80%) in our cohort being of the SCC subtype.

The optimal method of outlining cervical tumour volume on PET/CT remains contentious with various segmentation methods and thresholds described in the literature (Table 1). For pelvic malignancies, inclusion of adjacent high activity in physiologic structures (bladder, ureters and bowel) is particularly problematic requiring manual adjustment of the automated volume that has been mentioned but not fully documented by previous studies.

This study assessed three different segmentation methods to outline the cervical tumours: using percentage SUV_max_ thresholds with bladder masking when required (method 1), percentage SUV_max_ thresholds using isocontour method around the tumour prior to different SUV_max_ thresholds being applied (ellipsoid isocontour method, method 2), and an automated gradient method (method 3). This is the first study to assess inter-observer agreement of segmentation methods in cervical tumours and accurately document when any bladder masking and manual adjustment was required.

Our study has shown for method 1, MTV_25_ was closest to MRI volume for reader 1 and MTV_30_ closest to MRI volume for reader 2. For method 2, MTV_25_ had the closest correlation with MRI for both readers. Method 3 demonstrated a consistent technique that highly correlated between observers but significantly underestimated the MRI volume.

The Bland-Altman plots (Supplementary Fig. 3) demonstrated no significant difference only for reader 1 for method 1 at MTV_25_. All the other plots demonstrated proportional bias. The reason for this is that at extreme values, there was divergence between the MTV and the MRI values. This may be due to underlying extremes of SUV_max_ and/or the presence of necrosis (Supplementary Figs. 1 and 2).

The MTV_30_ threshold had excellent reproducibility between readers with narrow confidence intervals whilst MTV_25_ had moderate reproducibility with wider confidence intervals using method 1 but narrower confidence intervals on method 2 which permitted a constraining volume. Although the MTV_25_ was the only threshold to show no significant difference to MRI volume using paired *t* test for both readers using both pieces of software, this was at a trade-off of more requirement for manual adjustment using method 1 and thus reduced inter-observer agreement. Therefore, we propose that MTV_30_ offers the best combination of accuracy and inter-observer agreement along with less impact of the presence of necrosis and the extremes of SUV_max_.

Method 2 (ellipsoid isocontour method) had excellent correlation with MRI and excellent inter-observer agreement. However, it was not always possible to encompass the entire tumour without including bladder using the ellipsoid isocontour method. This method had a much higher correlation of above 0.75 for a number of different thresholds and overall the PET volumes were better correlated with the MRI volumes. This was due to manual adjustment not being feasible. Although we aimed to avoid manual adjustment in large tumours surrounded by bladder it was sometimes not possible to entirely exclude the bladder and only have tumour within the elliptic isocontour (Fig. 2). In future, if the constraining contour was not limited to a rigid ellipse, this method could be optimised further. The fact that no manual adjustment was performed on the VOIs was an added advantage because with method 1, even at the best MTV threshold, 44% required manual adjustment.

**Fig. 3:**
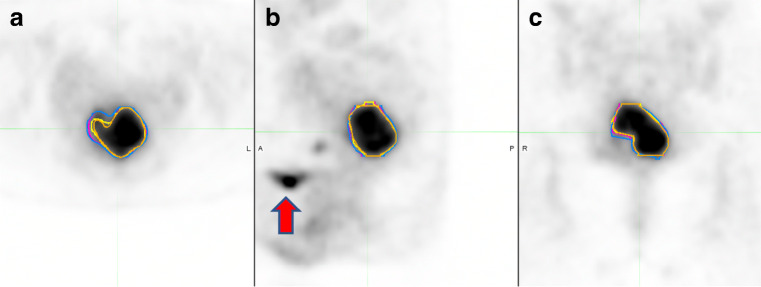
Method 3, the automated gradient method. Segmentation of the primary tumour, **a** axial, **b** sagittal, **c** coronal. The different colours show repeated attempts of segmentation from the same reader as an example. The bladder (red arrow) is far away from the primary tumour

Method 3 (automated gradient) was very simple to implement but required increasing adjustment for those that created segmentations which encompassed surrounding structures (Fig. 3). There was excellent inter-observer agreement but there was gross underestimation of the tumour compared with the MRI reference standard for the gradient method.

The gradient edge detection method identifies tumour based on a change in count levels at the tumour border. The gradient method evaluated in this paper calculates spatial derivatives along tumour radii then defines the tumour edge based on derivative levels and continuity of the tumour edge [30]. Compared to thresholding approaches, the gradient-based method better deals with the inherent shortcoming of PET images, such a low SNR and resolution. In phantom and surgical lung cancer studies, gradient-based methods have been proposed to best assess tumour volume compared to threshold methods [30, 38]. To the best of our knowledge, this is the first paper to compare threshold methods with a gradient method in cervical cancer. However, despite good correlations with the MRI volume the gradient method consistently underestimated cervical tumour volume. In lung cancers compared to background lung, the change in count level at the tumour border is more distinct. Whilst in cervical cancers the changes in count level at the tumour border may be less which could lead to underestimation. In addition, cervical tumours tend to have irregular rather than spherical shapes and it is possible this may lead to underestimation of the tumour. Currently, for this method, the MTV is generated by plotting two perpendicular orthogonal lines; however, in the future, this method will be optimised to take into account irregularly shaped lesions.

Traditionally, MTV_40_ has been used in the calculation of the MTV of cervical tumours based on a study by Miller and Grigsby [10]. This study, in only 13 subjects, suggested that MTV_40_ was the optimal threshold, using separately acquired CT images as a visual correlate. However, MRI, and not CT, is considered the gold standard for measuring cervical cancer tumour volume as cervical tumours are poorly demonstrated on CT [39]. In general, for individual tumours as the threshold lowers the measured metabolic tumour volume increases. In our study, use of the MTV_40_ led to a significant underestimation of tumour volume for both percentage SUV_max_ methods. However, at thresholds below MTV_30_, there was a higher likelihood of overestimating the tumour volume using PET.

As the MTV threshold is based on the SUV_max_, it was a concern that lesions with low uptake will have an overestimation of their metabolic volume and therefore a poorer correlation with MRI volume. Concordant with studies in lung cancer [40], we also demonstrated overestimation of the MTV in lesions with a low SUV_max_ most marked at MTV_25_ (Supp. Fig. 1).

Recent cervical cancer studies have independently explored the optimal MTV thresholds [13, 16, 17]. Upasani et al. in a study of 74 patients with stage IIB or IIIB squamous cell cervical cancer concluded that MTV_30_ and MTV_35_ were most optimal using tri-diameter ellipsoid based measurements of T2W MRI as the reference standard [13]. However, not all tumours are simple ellipsoid shape and this method may incorrectly estimate tumour volume in irregularly shaped tumours which may explain why they recommended a higher threshold compared to our study if MRI volume was potentially underestimated. Lai et al. evaluated 29 primary cervical cancer cases and as in our study, reported MTV_30_ to correlate best with MRI volume, which was measured by the same method as our study [16]. Manual adjustment was mentioned but not documented and inter-observer agreement was not assessed.

Cegła et al. assessed 30 cervical cancer patients and concluded that the MTV_35_ was the closest to the MRI reference standard; however, they did not detail the method of MRI volume measurement [17]. In this study, only three thresholds were evaluated and this limited the scope of outcomes. Using PET/MRI, Sun et al. [14] found that for their 35 subjects, there was no difference at the 35% or 40% threshold MTVs, T2W images and diffusion-weighted MR images. However, their numbers were small, and no mention was made of whether the tumour segmentations on PET encompassed the entire tumour, i.e. whether there were photopaenic regions due to cavitation, etc. In our study, 35 tumours had necrosis and 46 did not, and all areas were centrally located (Supp. Fig. 2). DWI is not established for accurate volume measurement, with limited reports in the literature and since it assesses tumour cellularity, it generates different measurements compared to T2 volume. The DWI volumes in their study were generally lower than the T2W MRI volumes whilst other studies have reported the DWI volumes to be generally higher than T2-weighted volumes [41].

Other studies have used a fixed absolute SUV_2.5_ [3, 25]. Although fixed thresholds can be useful in regions with very low background activity such as the lung, in the pelvis, a fixed threshold may include surrounding background structures and lead to overestimation of the tumour volume. In our study, the fixed SUV_2.5_ led to 69.7% overestimation of tumour volume when compared to the MRI volume and required the most manual adjustment (Table 3) using method 1 due to the inability to use a constraining volume with this method. The situation was markedly improved, however, using method 2, where the isocontour permits a restrained volume (percentage overestimation of the tumour volume 27.6 for reader 1 and 22 for reader 2). Our findings are consistent with Zhang et al. who reported SUV_2.5_ overestimated cervical tumour volume (based on T2-weighted MRI) in the majority of cases and concluded it was unsuitable for thresholding of cervical tumours [15].

Bladder masking overcame one of the reasons previously cited for not using lower SUV_max_ thresholds for tumour volume estimation [10] (Table 2). For method 1, overall 86% had bladder masking and the requirement was greater at lower thresholds (93% required bladder masking at MTV_25_, 89% at MTV_30_ and 84% at MTV_40_). Other studies have mentioned the use of this technique but have not mentioned the frequency of its usage [12]. This is the first study to accurately document the requirement for bladder masking and manual adjustment. Bladder masking was not available for method 2 and for method 3, only 4% required bladder masking. In our study, one observer performed the bladder masking for method 1 but as the masking was automated, this was unlikely to impact on the inter-observer variation.

All methods have their strengths and weaknesses. Ideally, the method of MTV delineation should be accurate, easy to use and reproducible. Therefore, as automated as is feasible but will depend on locally available software. In addition, readers should be aware absolute MTV measurement can vary with the software method available.

High-resolution T2-weighted sequences are recognised as the gold standard for tumour outlining by GYN GEC-ESTRO working group guidelines for cervical cancer brachytherapy tumour outlining [31]. The MRI based tumour volume technique used in our study (multiplying the sum of the tumour areas by the slice thickness) is considered the standard MRI volume technique closely correlating with gross specimen [32]. In our study the MRI volumes were generated by a single experienced observer; however, using the same method, Dimopoulos et al. [42] demonstrated acceptable inter-observer variability from two independent observers. In addition, manual segmentation of the primary tumour using individual slices is more accurate than using three orthogonal measurements of the tumour to compute the volume of an ellipsoid as most cervical cancers are not ellipsoid [43]. Using volumetric based MRI measurement, the MTV_25_ correlated closest with the MRI volume for reader 1 and MTV_30_ for reader 2. As mentioned earlier, studies using 3 orthogonal measurements suggested MTV_30_ and MTV_35_ correlated best with MRI volumes [13]. Lau used a similar method to this study but averaged the sagittal T2W volumes obtained by two readers and found that MTV_30_ was the closest to the MRI volume [16].

Although radiotherapy planning is based on MRI volume, due to the excellent depiction of patient anatomy and dose constraints to normal structures, there is a role for PET in patients unable to have an MRI and there may be a role of PET alongside MRI for auto-contouring of tumours for radiotherapy planning. In addition, the volumetric data derived from the MTV can be further assessed in radiomics studies in order to predict prognosis and evaluate the future success of adjuvant therapy.

Partial volume effect (PVE) may also influence the PET volume calculation, particularly for small tumours. Whether PVE leads to over or underestimation of MTV depends on target to background ratios (TBR). More avid tumours with higher TBR size may be overestimated and those with lower TBR may be underestimated [44]. In our study, we like other groups [11, 14] excluded small tumours < 5 cm^3^ due to the PVE. MR volume is less susceptible to PVE due to the higher spatial resolution.

A limitation of our study was in some cases mainly MTV_20_ for method 1; the automated volume included a lot of normal structures or physiologic activity (sometimes even extending along ureters to kidneys and including the heart) and were deemed ‘too difficult’ to manually adjust; thus, MTV was not documented. This could lead to bias; however, it involved very few cases (for method 1: 2 at MTV_30_, 6 at MTV_25_, 7 at MTV_20_; method 3: 1 for each reader), (Fig. 1 and Table 4). We would propose, in clinical practice, if the MTV_30_ was too difficult to manually correct then select MTV_35_ instead.

Although there were two observers for each method, the second observer was different for method 1 and the level of clinical experience of the observers was different (15 years versus 3–5 years). However, regardless of the difference in the level of clinical PET/CT experience, since MTV is not routinely performed clinically, all observers received the same software training prior to the study. In addition, there was consistently good-excellent inter-observer agreement across all methods suggesting the years of clinical experience did not seem to impact the output.

The time taken for the segmentation has only been briefly discussed in the literature [45]. Although the time taken for outlining using method 1 and 2 was not accurately recorded, the former took a lot longer, approximately 15 min per scan, compared with 5 min per scan for the latter. The time taken for each scan for method 3 varied greatly from 5 min for the quick scans that required no adjustment to up to 20 min for the more demanding scans.

Another limitation of our study was that we used a correlation method to compare the PET and MRI volumes. The volume does not demonstrate that the tumour volumes obtained from the two modalities match or overlap. A method to overcome this is to use the DICE method [46] or similarity coefficient that measures the degree of overlap [27]. However, due to the effect of bladder filling changing the position of the tumour, it may not be possible to use this method to truly compare the segmentations from different modalities. Using DICE on the same modality is definitely a more accurate method and creating masks for all the PET images would be a useful area of work.

In PET/MRI, when the PET and MRI images are obtained contemporaneously, there may still be some difference in the appearance of the tumour between the two modalities due to variable bladder filling in the time interval between acquisition. The few studies [14, 47, 48] that have used PET/MRI for volume have stated that there was excellent co-registration between the two modalities, with the caveat that no mention of bladder filling was made. Perhaps simultaneous acquisition improves the degree of overlap between the two modalities.

All the FDG PET/CT analysis was performed with the same reconstructions on retrospective data from the same scanner. Two other studies [13, 16] from other centres using different PET manufacturers (GE Discovery VCT) and reconstruction parameters also demonstrated the same optimal threshold. The effect of resolution recovery on the MTV has not been explored but as this method of reconstruction becomes more common, this may impact on the optimal segmentations.

A recent radiomics study recognised that MTVs connecting bladder is a major problem for most segmentation methods and utilised MTV 50% to avoid bladder at the trade-off of under-sampling tumour volume [49]. A systematic review and meta-analysis, reported MTV and TLG were significant prognostic factors in patients with cervical cancer [5] in spite of different methods of outlining. Future work should assess if the MTV threshold/ method within the same patient group has a different impact on predicting outcome/radiomics.

The widespread adoption of MTV will rely on the ease of use and reproducibility between observers. Future software development may permit selection of constraining volume (as in method 2) but in addition, the ability to slightly adjust the constraining volume for such cases where the tumour and bladder cannot be entirely separated by the isocontour method.

## Conclusions

In conclusion, for tumours > 5 cm^3^, MTV_30_ provides the optimal correlation with MRI volume taking into consideration the excellent inter-reader agreement and less requirement for manual adjustment along with less impact of the presence of necrosis and the extremes of SUV_max_. Depending on local software method for MTV outlining, masking of bladder activity or the use of a constraining volume prior to auto-contouring enables volume measurement at lower SUV thresholds without inadvertent inclusion of bladder activity.

## Supplementary information

ESM 1 (DOCX 512 kb).
