# Supplementary material for: Optimal method for metabolic tumour volume assessment of cervical cancers with inter-observer agreement on [18F]-fluoro-deoxy-glucose positron emission tomography with computed tomography
Source: Eur J Nucl Med Mol Imaging. 2020 Dec 11;48(6):2009–23. doi: 10.1007/s00259-020-05136-8 (PMC8113292; doi:10.1007/s00259-020-05136-8)
Supplement: Supplementary file 1 — (DOCX 512 kb). [file 259_2020_5136_MOESM1_ESM.docx]

(a)

(b)

Supplementary Figure 1 The mean percentage difference between selected MTV thresholds at low, intermediate and high SUV_max_ compared with the MRI volume for both readers for (a) Method 1 and (b) Method 2.

(a)

b)

Supplementary Figure 2 The mean percentage difference between selected MTV thresholds with necrosis or not compared with the MRI volume for both readers for (a) Method 1 and (b) Method 2.

(a)

(b)

(c)

(d)

Supplementary Figure 3. Bland Altman plots compared with MRI for (a) Method 1, Reader 1, (b) Method 1 Reader 2, (c) Method 2, Reader 1, (d) Method 2, Reader 2.
